# Enhanced Human Activity Recognition Based on Smartphone Sensor Data Using Hybrid Feature Selection Model

**DOI:** 10.3390/s20010317

**Published:** 2020-01-06

**Authors:** Nadeem Ahmed, Jahir Ibna Rafiq, Md Rashedul Islam

**Affiliations:** 1 Centre for Higher Studies and Research, Bangladesh University of Professionals, Mirpur Cantonment, Dhaka-1216, Bangladesh; nadeem11@gmail.com; 2 Department of Computer Science and Engineering, University of Asia Pacific, 74/A, Green Road, Dhaka-1205, Bangladesh; jahir@uap-bd.edu; 3 School of Computer Science and Engineering, University of Aizu, Fukushima 965-8580, Japan

**Keywords:** human activity recognition (HAR), feature selection, machine learning, SVM, sensor, accelerometer, gyroscope

## Abstract

Human activity recognition (HAR) techniques are playing a significant role in monitoring the daily activities of human life such as elderly care, investigation activities, healthcare, sports, and smart homes. Smartphones incorporated with varieties of motion sensors like accelerometers and gyroscopes are widely used inertial sensors that can identify different physical conditions of human. In recent research, many works have been done regarding human activity recognition. Sensor data of smartphone produces high dimensional feature vectors for identifying human activities. However, all the vectors are not contributing equally for identification process. Including all feature vectors create a phenomenon known as ‘curse of dimensionality’. This research has proposed a hybrid method feature selection process, which includes a filter and wrapper method. The process uses a sequential floating forward search (SFFS) to extract desired features for better activity recognition. Features are then fed to a multiclass support vector machine (SVM) to create nonlinear classifiers by adopting the kernel trick for training and testing purpose. We validated our model with a benchmark dataset. Our proposed system works efficiently with limited hardware resource and provides satisfactory activity identification.

## 1. Introduction

Detecting, interpreting, and identifying human movements during different activities such as walking, running, eating, lying down, sitting down, and so on are generally considered human activity recognition (HAR). Human activity recognition has far-reaching significance due to the fact that it is the primary requirement for many useful applications such as healthcare [1,2], surveillance activities [3,4], context-aware computing [5,6], athletics [7], and smart homes [8]. In the medical industry, accurate detection of human movement has the potential to develop correct diagnosis autonomous system using machines, while detection of running and walking can help video surveillance, smart homes, and similar activities.

Human activity recognition lies at the heart of numerous automation projects that make our lives easier. From motion sensor-based lights turning on by affirmatively identifying the presence of a user to modern high-tech facilities that even provide separate services to separate users based on their facial features such as fingerprint and retina. Not only will this make our lives more convenient, but it will also make our lives safer and save our precious time.

Human activities were mostly investigated by sensor-based systems such as accelerometer and computer-vision based systems. While the former is not always authentic due to the fact that sensor positioning is not fixed [9], the latter suffers due to motion blur, angel, obstruction on the path, and varying illumination conditions in environments that are generally sophisticated [5]. Among these two methods, sensors are preferable to researchers due to a number of merits including its lightweight nature, being easily portable, mountable at different locations, withstanding low-power, relatively low energy usage, and so on. However, we have concluded that without a very efficient classifier, data from sensor can be misleading.

Smartphone-based sensors are gaining popularity as this particular method has proved to be the equilibrium between data accuracy and usability of the procured data. Many applications rely on constant feeding on data from their ipso facto source such as elderly people in their homes under observation. This is true for not only for elderly care, but also all for sorts of applications, which follow the loop: Observe the particular object of interest; calculate the level of deviation from the standard point set out at the beginning; take action based on the deviation; run the loop again. However, sensors can only provide raw data and they vary and attenuate from object to object, time to time, and therefore, a great deal of emphasis is put on accuracy before any concrete measure is taken.

Since wearable technology such as wristbands is commonly used for various reasons—including easier communication, portability, and trend—researchers can easily capitalize on the techy trend. This technology is truly ubiquitous in nature and the sensors used in these devices can provide valuable data as far as that particular user is concerned. A pool of such data shall provide a basis for features of one particular activity such as walking. Similarly, jogging and its feature can be extracted and the same goes for other types of activities. A user-centric application can provide a much better service should it understand the user on a root level.

However, researchers have encountered a problem with the position of a wristband. For instance, a recent study clearly showed that a sensor fitted on the waist was successful in identifying singular, one-dimensional activities such as walking or sitting down on a chair while it struggled to identify combinational activities like eating or driving a car [10]. We need to fully understand each activity to reach a conclusive point, and therefore it is more conducive when we can identify activities outside of test conditions.

Many human activity identification researches had been conducted based on video data of activity. These researches can be largely classified in the field of image processing. However, using image as a way to identify different activity has a major drawback i.e., it violates the privacy of users. In many applications, it can be regarded as a significant problem. For example, monitoring the elderly people in a care home, we cannot simply install cameras in every corner to monitor the activities of senior citizens. It will cause a major privacy issue. In this regard, sensor-based technology has brought relief in such applications. The main idea behind a sensor-based technology is to mount wearable devices in human body. Wearable devices are equipped with sensors that can produce data based on human orientation. Accelerometers and gyroscopes are the most widely used sensors for activity identification; generally, they are incorporated in wearable devices such as smartphones, smartwatches, fitness trackers, fitbits, and so on. Generally, smartphone inertial sensors generate data chronologically in the form of a time series.

Machine learning requires a very large number of data, especially when the dimension of the data increases significantly, the data required for an accurate analysis increases dramatically. “Curse of dimensionality” as termed by Bellman [11] is an important aspect to consider because sensor-based systems provide a lot of data. Therefore, it is important to select certain patterns or features, and based on the features, we can classify. As the number of data rises, the computational cost also rises exponentially. That is why we need to use feature subset selection and feature extraction.

In this paper, we have emphasized continuous data procurement using smartphone-based sensors such as a gyroscope and accelerometer. Sensors such as accelerometers and gyroscopes produce a total of six-axial data for every activity. All sensors are important to figure out the activities. Features have been calculated on these axial data. For reducing the probability of missing information, a high-dimensional heterogeneous feature vector is extracted from the time-series data. Though there are 23 original features, a total 138 features are extracted from six-axial sensor’s data for identifying the activity. However, every feature of the total feature vector is not equally important. Some features may be irrelevant and some are redundant. Moreover, such a huge number of features is an overhead for classification purpose. Thus, a new hybrid optimal feature selection process is proposed. This proposed hybrid feature selection methodology is a proper combination of filter and wrapper approaches of feature selection. Based on the feature selection, an optimal feature vector is generated in the feature analysis section, which was used in the classification process during human activity recognition using SVM. We have divided our data into two parts: Test dataset and trained dataset. Here, a synthetic dataset is used to test the authenticity of the technique proposed here. Our main contributions here are:

1. Human activity recognition using motion sensor data of smartphone.
2. A hybrid feature selection model combination of SFFS based filter approach and SVM-based wrapper approach.
3. An effective human activity identification using multiclass support vector machine.

The full paper is organized as follows. The next section discusses previous work and their outcome; then, the section three discusses the required methods and necessary equipments. The section four provides the experiment results and discussions. In section five, additional experimental studies were included. Lastly, the section six concludes this research with future scopes. In every section, necessary figures, tables, and graphs are included for easier understanding and clearer explanation.

## 2. Literature Review

Gyllensten et al. proposed a classification algorithm based on machine learning to identify a range of activities including walking, running, sitting down, lying down, and cycling [12]. While the researchers noted that under laboratory conditions, the outcomes were generally acceptable, the performance in free-living condition i.e., untrained data would not produce satisfactory results. The limitation, as we note, is that continuous movement tracking could be implemented using sensors, preferably body-mountable, to better understand the activities of their agents.

Esfahani et al. showed that by placing multiple sensors at carefully selected places on the human body, the precision of classifying the activities of the subjects goes up significantly [13]. The work included collecting data from the subjects and then placing them in a pool to check where they belong, it would be a massive problem to detect complex activities due to the fact that a separate model would be needed; most untrained data would not be classified as expected.

In computer vision, video surveillance of objects relies mostly on support vector machines and its classifiers and piece-wise linear support vector machine (PL-SVM) [14]. The technique is widely used to interpret and reconstruct from two-dimensional views, since it can easily provide valuable insight into any moving object. This paper demonstrates the need to succinctly utilize this property while the major drawback is this will not easily provide solution where we need to identify the activities more precisely.

Human activity can be detected using smartphone sensors as they are omnipresent and provide easy access to useful data. Ahmed et al. showed in their investigation that smart sensors are better equipped than conventional video streaming [15] and analyzing as they are often real-time in nature. Although smartphone sensors are quite handy, the performance analysis by Chen et al. is proof that the raw data should be handled more technically to generate an amicable output [16]. That is the reason that in our investigation, we have emphasized more the use of efficient hardware-based classifier, divining the date in trained and test data; extracting features so as not to leave a gap between performance and sensors data.

Mohammed Mehedi Hassana et al. collected physical human activities by using wearable sensors in a smartphone in three-axis signal of an accelerometer and gyroscope. After extracting features from raw data so as to increase their robustness, features are then processed by kernel principal component analysis and linear discriminant analysis. Finally, features are trained with a deep belief network (DBN). Then, DBN result was compared with traditional expression recognition approaches such as typical multiclass support vector machine (SVM) and artificial neural network (ANN). They reached up to 95.85% accurate results on the test set [17].

RubénSan-Segundoa et al. collected data from smartphone and smartwatch in three-axis signal of an accelerometer and gyroscope. Extracted features from the raw data were applied two methods: CNN and HMM. Accuracy for smartwatch improved from 78.6 to 88.1 and for smartphone improved from 96.4 to 98.1. This is clear proof that a feature normalization technique is quite helpful for smartphone data but not helpful for smartwatch data [18].

Ran Zhu et al. gathered data from three different sensors (accelerometer, gyroscope, and magnetometer). The dataset consisted of 100 subjects’ sensory samples. They processed the dataset based on a convolutional neural network and obtained a reasonably acceptable result of 95.62% accuracy, while processing the dataset using novel ensembles of CNN returned an accuracy of 96.29% [19].

Tsige Tadesse Alemayoh et al. used smartphone (accelerometer and gyroscope motion sensors’ signal) and converted these data into 14 × 60 virtual image. Next, they developed an iOS application for recording and streaming motion data; it was further used to identify real-time activities. For classification, those processed data were given to the designed convolutional neural network. The time-series data structure can generate better results in terms of accuracy for predicting activities. Overall, they obtained an accuracy of 99.5% [20].

Li Fang et al. extracted features from raw data and processed them using support vector machine (SVM), K-nearest neighbor, and logistic regression. Average accuracy for SVM was 88.9%, KNN was 95.3%, and logistic Regression was 83.9%. The paper mainly focused on up and down buses. For these two activities, accuracy was 89.5% for SVM, 96.8% for KNN, and 89.7% for LR for up bus, and for down bus, 84.2% for SVM, 94.2% for KNN, and 81.9% for LR [21].

Yu-Liang Hsu et al. obtained motion signal from two inertial sensing modules, which subject wore on their wrists and ankles, based on 10 daily activities. Nonparametric weighted feature extraction algorithm were used to decrease the feature dimensions for processing raw data. Classification of the data was done using probabilistic neural network, where the accuracy rate is 90.5% [22].

Xizhe Yin et al. utilized four machine learning methods (J48, support vector machine, naive Bayes, and multilayer perceptron) for detecting three basic activities and two activities. The processing step was divided into four subject areas. Firstly, a smartphone-based approach; secondly, data collection; thirdly, feature extraction; and finally, classification. Data were collected in a three-axial accelerometer, three-axial linear accelerometer, gyroscope, and orientation. J48 is an algorithm, in which the output is produced in a decision tree and accuracy is 96.8%. Accuracy of others based on five activities is 98.7%, 97.8%, and 98.5%. The accuracy of all methods is 99% + when using only three basic activities. Finally, they concluded J48 is more efficient because it is easy to compute and can be converted into IF-THEN rules. Other classifiers method can produce satisfied results [23].

Ankita Jain et al. used UCI HAR dataset and physical activity sensor data in their research. Their process steps are (1) input signals, (2) extract additional signal, (3) feature extraction, (4) information fusion, (5) classification. THE Accuracy of feature level fusion SVM is 97.12% and K-NN is 91.75%. The accuracy of score level fusion SVM is 96.44% and K-NN is 84.02% [24].

Yiyuan Chen et al. proposed an important classification method that uses a binary particle swarm optimization (BPSO) technique for optimum feature selection. Two key areas are explored to improve efficiency for classification, namely confidence of the feature and lower cost of these features by considering the relationship between features, while their categories are considered for feature cost and reduction ratio [25]. This will significantly improve data classification, especially for healthcare.

However, accuracy of activities and computational cost are outstanding issues in the HAR-related work since limiting one pushes the other above expected level. Liang Cao et al. proposed a solution to this problem by introducing a method that takes the context under consideration and groups the activities to reduce error and complexity of computation [26].

## 3. Proposed Model

**Figure 1:**
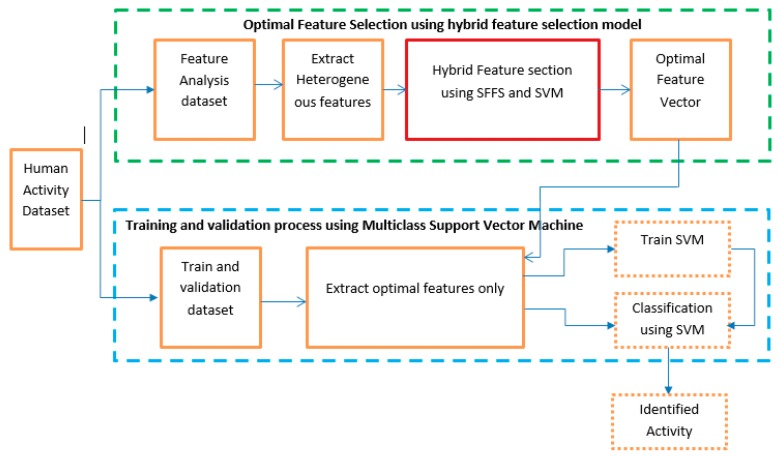
Structure of the proposed model of activity identification.

In this section, the proposed model of activity identification is briefly described in a step by step procedure. A block diagram of human activity identification is shown in Figure 1. The model is basically divided into two major parts. The first part depicts our main contribution in the experiment. The first part consists of selecting the optimal feature sets from the dataset. The dataset is split into two equal subsets. The first subset is used to generate optimal features using a hybrid feature extraction process. Several statistical features are calculated from three-axis accelerometer and gyroscope signals from the first data subset. Both time-domain and frequency-domain statistical features are considered in the model for better accuracy. We have used a sequential floating forward search (SFFS) feature selection algorithm in this proposed model. In the second procedure, validation is completed using the second data subset. A machine learning technique is deployed to identify the human activity based on the selected feature set. We have used multiclass support vector machine (SVM) in this research.

### 3.1. Activity Data Collection

Human activity recognition is a popular research interest amongst many researchers. Activity data are widely available in different repositories. Accelerometer and gyroscope are the two most common sensors that are used for human activity identification. Accelerometer and gyroscope data, for example, are represented by a set of three vectors *acc_i_ =* (*x_i_*, *y_i_*, *z_i_*) and *gyro_i_ =* (*x_i_*, *y_i_*, *z_i_*), where *i =* (1, 2, 3, …, n).

An accelerometer is a compact device designed to measure non-gravitational acceleration. When an integrated accelerometer goes from a standstill to any velocity, the accelerometer is designed to respond to the vibrations associated with such movement. It uses microscopic crystals that go under stress when vibrations occur, and from that stress, a voltage is generated to create a reading on any acceleration. Accelerometers are important components to devices that track fitness and other measurements in the quantified self-movement.

A gyroscope is a device that uses Earth’s gravity to help determine orientation. Its design consists of a freely rotating disk called a rotor, mounted onto a spinning axis in the center of a larger and more stable wheel. As the axis turns, the rotor remains stationary to indicate the central gravitational pull, and thus, which way is “down.”

### 3.2. Heterogenious Statistical Feature Extraction

Statistics is a robust instrument that can operate on data and produce meaningful information. It can produce technical data by which data analysis is possible from high-level perspective. From a high-level view, statistics is the use of mathematics to perform technical analysis of data. Statistics is capable of producing both visualization graphs and in-depth statistical data, which are more information-driven and work in a targeted way to reach concrete conclusions about our data rather than just projecting. Thus, statistics creates deeper and fine-grained insights, which exactly depict the data structuring. Utilizing other data science techniques side by side optimally helps to produce even more meaningful and in-depth information.

Feature is a statistical function that works brilliantly to extract meaningful information of data in a natural way. From the perspective of human activity recognition, a particular pattern is generated from a particular physical movement of users. As an example, the “run” activity has a particular pattern as it involves superior physical effort from a human, which is quite different from the “walking” activity pattern. Thus, inertial sensors like an accelerometer and gyroscope can measure the intensity of each physical effort and produce different pattern distributions. Collected pattern distribution i.e., statistical data information from these sensors can distinguish between “walking” and “running” activity. Hence, the standard deviation or any other statistical feature is capable of highlighting the difference between these two activities.

In the feature extraction process, we extract the time and frequency domain statistical feature from three axial data from both accelerometer and gyroscope. To extract the maximum information, a total of 23 base features have been applied to collected data, which includes both time domain and frequency domain statistical parameters. The 23 features, including their corresponding statistical formulas, are formulated in Table 1.

### 3.3. Feature Selection

All features are not equivalently contributed in activity classification. Inclusion of every single feature creates multiple dimensions and leads to an overfitting problem. To avoid the overfitting caused by extraneous dimensions, we have applied a smart feature selection procedure to identify the important features. It is necessary for the learning algorithm to focus on the relevant subset of features and ignore the rest of the features. The particular learning algorithm works on the training set to select the best performance feature subset, which can be applied to testing set. For yielding a higher classification outcome, this research applied a hybrid approach of feature selection to lower the dimensionality of the original feature space [27].

Our described proposal consists of two parts: A filter-based approach using sequential forward floating search (SFFS), in which the feature subset evaluation process is independent of the classification algorithm and a wrapper-based approach, which evaluates the best feature set amongst all the feature sets that were derived from filter-based approach.

**Figure 2:**
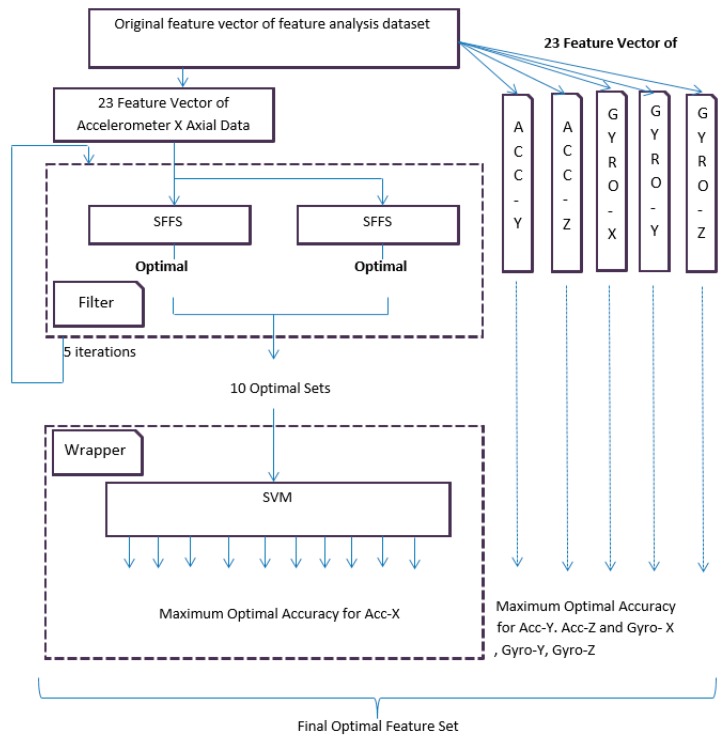
Overall block diagram of the proposed hybrid feature selection.

The filter-based feature analysis is conducted by SFFS in an iterative way. At first, 30 users’ first datasets are dedicated for training and testing purposes and the same 30 users’ second datasets are dedicated for validation process. The dataset for training and testing is again split into two equal parts in a random process. The first equal part is further divided in two equal parts and used in a filter-based approach. The second equal part is used for testing in wrapper approach. SFFS is applied in the first equal part where the process iterates for five times and yields 10 best optimal sets. SFFS works as an evaluation function (fitness function) to select the suboptimal features. The same procedure is also applied to all other sensors signal. These optimal feature sets use the wrapper method. Wrapper analyzes different optimal sets by using support vector machine to evaluate the best feature set. The whole process is depicted in Figure 2.

In the filter-based approach, SFFS is used to find the optimal sets. Among the most satisfactory feature choice scheme for wrappers is the SFFS [28]. SFFS is first used to produce a series of feature subsets. Then, a discriminatory feature subset candidate is determined among them, where the feature subset candidate can be insensitive to outliers. That is, to successfully discriminate the feature subset candidate, proper assessment of feature subset quality is a key issue. Our study mainly focuses on SFFS. It can accurately yield a sequence of quality feature subsets. After that, we determine inequitable feature subset candidates. Algorithm 1 of SFFS is described in the following:


**Algorithm 1**
Input: the set of all features $Y=\left\{y_{1},\;y_{2},\;\ldots ,Y_{n}\right\}$
Output: a subset of features $X=\{\;x_{j}|\;j=1,2,3,\ldots ,\;k;\;x_{j}\in Y\}$
Where, k=(0,1,2,…,n)
Steps:
1. $Y_{0}=\left\{\emptyset \right\}$
2. Select the best Feature X^+^
Update: $Y_{K+1}=Y_{K}+X^{+};=\;+1$
3. Select the best Feature X^−^
4. If $J\left(Y_{K}-X^{-}\right)>J\left(Y_{K}\right)$    [(X) = Criterion func.]
Then, $Y_{K+1}=Y_{K}-X^{-};K=K+1$
go to step 3.

In the wrapper-based approach, support vector machine is applied on the second equal part of the dataset, which is dedicated for training and testing purposes. Therefore, the 10 optimal sets that are gained from the filter-based approach are fed to the support vector machine. It is necessary to measure the best feature set from out of the 10 optimal sets by using SVM. In Figure 2, the process is shown for the accelerometer *x*-axial data. In the same way, the whole process is also applied for accelerometer *y*, *z* axial data, and gyroscope *x*, *y,* and *z*-axial data.

### 3.4. Objective Function Based on Discriminant Feature

**Figure 3:**
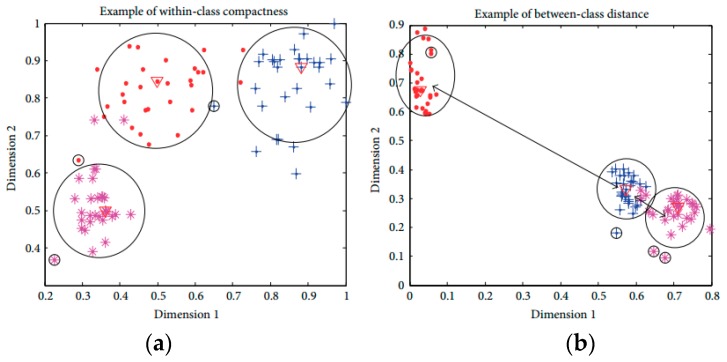
Examples of exceptions in feature distribution. (**a**) Classes are well-separated; (**b**) Classes are overlapped.

In this research, the distribution of activities is generally high-dimensional complex. Sometimes, the average Euclidean distance is not capable of fully describing the sample distributions based on within-class compactness and between-classes separation. The mean distance based compactness is not capable of managing all possible sample distributions as shown in Figure 3. All class samples are distributed in a way that two classes are overlapping, with one class is positioned at a far distance. The large distance values can lead to inexact inferences as to the distribution of samples using the average distance-based interclass separability. To deal with this problem, this research introduces a discriminant feature distribution analysis-dependent objective function.

In objective value calculation, firstly, the Euclidean distance metric is used for calculating the distance between two samples in the *n*-dimensional feature space. If *x* and *y* are two points with *n*-dimensional features, the distance calculation can be formulated as follows:

$$\begin{array}{l} d_{x,y}=\sqrt{{\left(x_{1}-y_{1}\right)}^{2}+{\left(x_{2}-y_{2}\right)}^{2}+\ldots +{\left(x_{n}-y_{n}\right)}^{2}}\\ \begin{array}{ccc} & & =\sqrt{\sum_{i=1}^{n}{\left(x_{i}-y_{i}\right)}^{2}} \end{array} \end{array}.$$

Based on the distance between all samples of a class, the class median is determined. The class media is *i*-th data sample, which has minimum accumulated distance satisfying $i=\arg\min\{D_{cumulated}^{i}\}$ with other samples in the class. The calculation of the cumulated distance of a data sample is formulated as follows:

$$D_{cumulated}^{i}=\sum_{j=1}^{n}{\left\|x_{i}-y_{j}\right\|}_{2},\begin{array}{cc} & i=1,2,\ldots ,n \end{array}.$$

**Figure 4:**
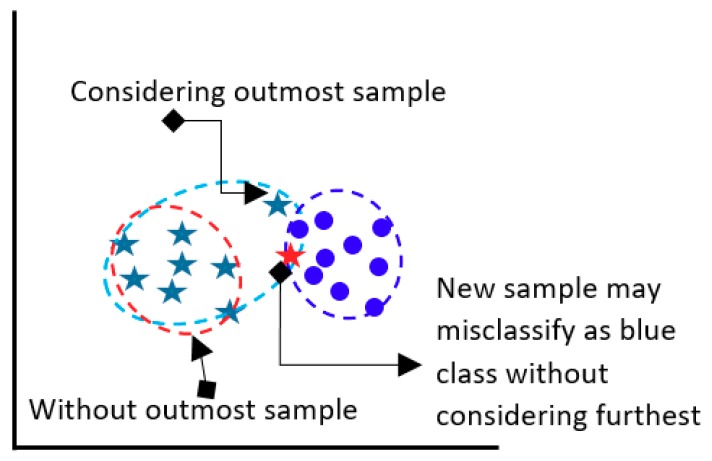
Consideration of outmost sample of a class.

The model determines the Euclidean distance between class median point and farthest point as a within-class compactness value for that class. The farthest sample of a class might be a bad sample, however that sample is a member of class and should be considered. If the furthest sample is ignored, then the overall class boundary will be reduced and a new sample may be misclassified in future classification. Figure 4 presents the above situation.

The two between-class distance is understood by the boundary points of those classes. Normally, it is measured by the minimum value of the Euclidean distances from all the available samples of remaining classes. The ultimate target of discriminant feature distribution analysis is to make the objective function maximize. The objective function is maximized with minimal within-class compactness and maximal between-class distance. The measured objective value of the optimal features guarantees the best distribution of samples of distinct activities, which ultimately increases the classification outcome. The formula below calculates the objective value:

$$objective\_value\;=\;\frac{between\_class\_distance}{within\_class\_compactness}.$$

**Figure 5:**
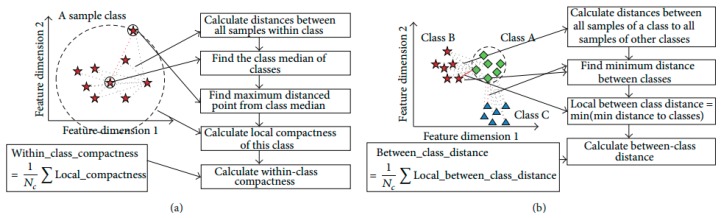
(**a**) Within-class compactness value and (**b**) between-class distance value calculation.

Figure 5 explains the processes of objective value calculation by measuring within-class compactness and between-class distance. The class evaluation and distances calculation are formulated during feature analysis and optimal feature selection for a dataset, which might take large computation; however, it helps to find the optimal features and discard the irrelevant and redundant features. The optimal features are used in classification of activity on fly with less overhead on classification model.

### 3.5. Acitivity Recognition for Validation Purpose

**Figure 6:**
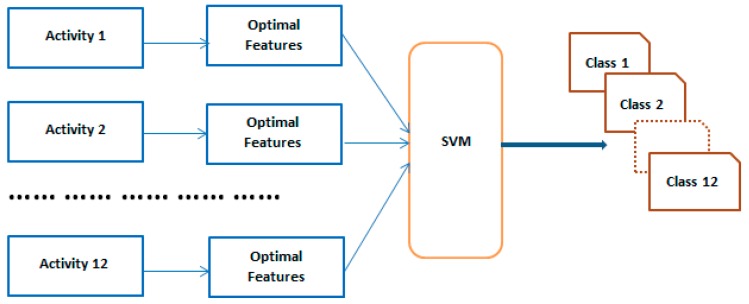
Overall block diagram of the validation process.

The filter and wrapper approach ultimately have optimal features, which work best in the 30 users’ first dataset. In this validation state, optimal features are validated against the rest of the data to the check the robustness of the model. In the online process, the one-against-all support (OAASVM) classifier is also used [29] for multi-faults classification with linear kernel function to validate our selected feature sets in terms of the classification accuracy. The SVM with linear kernel function classifier is one of the most popular classification methods and is widely used due to simplicity and computational efficiency. Figure 6 shows the overall validation process.

## 4. Experimental Results

**Figure 7:**
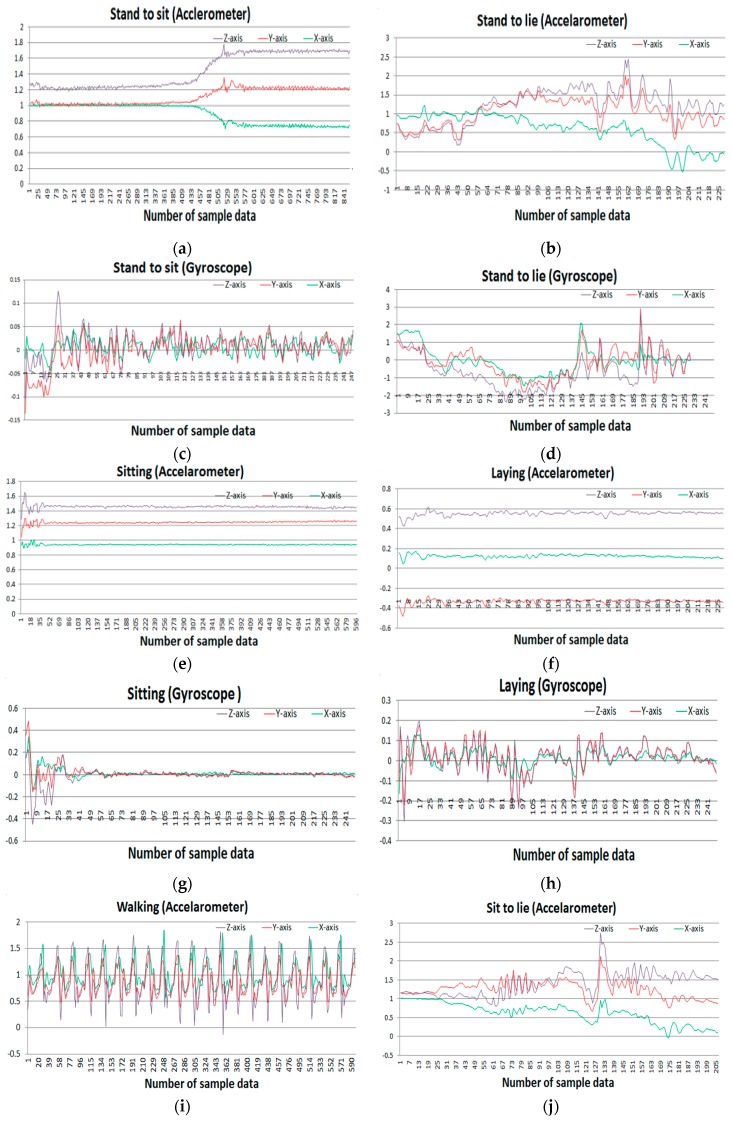

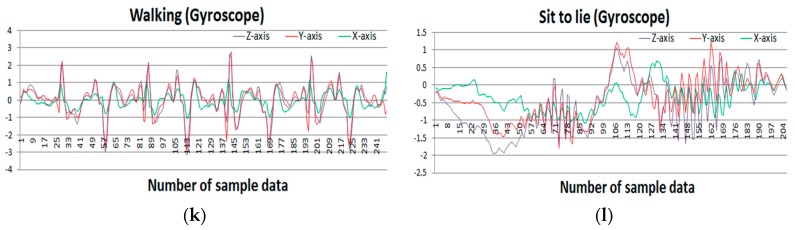
Sample accelerometer data (**a**,**c**,**e**,**g**,**i**,**k**) and gyroscope data (**b**,**d**,**f**,**h**,**j**,**l**) for different activities: (**a**,**c**) Stand to sit activity, (**e**,**g**) sitting activity, (**i**,**k**) walking activity, (**b**,**d**) stand to lie, (**f**,**h**) laying activity, (**j**,**l**) sit to lie activity.

This public dataset is gathered from UCI machine learning repository [30]. A group of 30 people with an age range of 19–48 years were selected for data generation in this experiment. They performed 12 activities in different time settings. The recorded activities are stationary, dynamic, and a combination of both. The stationary activities are standing, sitting, lying; the dynamic activities are walking, walking downstairs, and walking upstairs; and combined activities are stand-to-sit, sit-to-stand, sit-to-lie, lie-to-sit, stand-to-lie, and lie-to-stand. People in the experiment were given a mountable Samsung Galaxy S II smartphone on their waist. This smartphone has multiple sensors including accelerometer and gyroscope, which were used to generate a time series of data for the experiment. Both sensors generate three-axial data series depending on the orientation of the object. The researchers conducted a video recording on the whole experiment manually and set the time interval fixed at 50 Hz. Figure 7 shows the sample accelerometer and gyroscope data for different activities.

In the experiment, there are total 138 original feature vectors. There are main 23 features and dual three-axial data of accelerometer and gyroscope. These feature vectors are directly fed to the filter process of optimal feature selection. Table 2 provides an individual number to every feature with its corresponding sensor axis.

In filter-based approach, these feature vectors are executed by two individual SFFS algorithms on two randomly split datasets. Both datasets contain equal numbers of data. Individual SFFS produces separate optimal feature sets on both datasets. Again, this whole process is iterated for another four times. As the dataset is divided on a random basis, each iteration produces separate optimal feature sets. Thus, the filter process produces 10 optimal feature sets for each of the axial data i.e., a total 60 sets are produced for all axial datasets.

**Figure 8:**
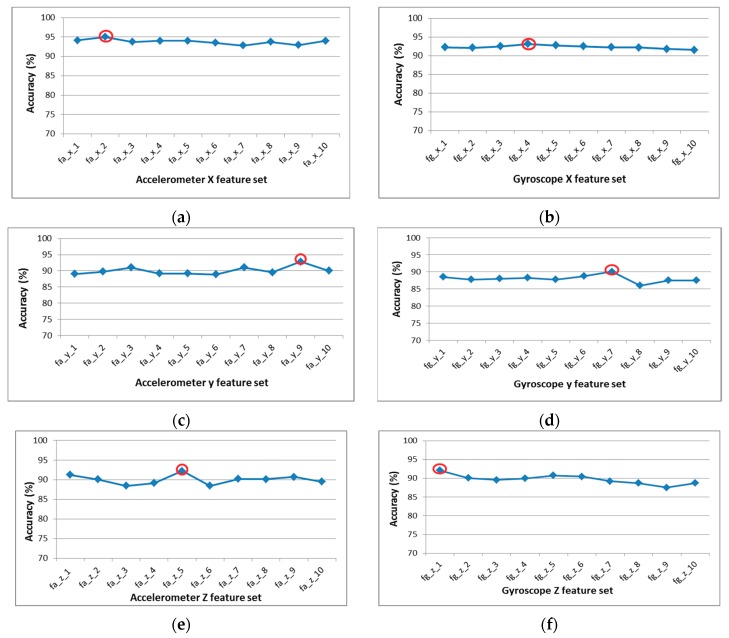
Accuracy of different feature sets produced by support vector machine in wrapper method. Highest accuracy is mark in red circle: (**a**) Accelerometer X-axial feature set; (**b**) accelerometer Y-axial feature set; (**c**) accelerometer Z-axial feature set; (**d**) gyroscope X-axial feature set; (**e**) gyroscope Y-axial feature set; (**f**) gyroscope Z-axial feature set.

The 10 sub-optimal feature sets are passed through the wrapper procedure, where the classification accuracies are calculated using SVM for each sub-optimal feature vector. Figure 8 presents the classifications accuracies of all sub-optimal feature vector of six-axial data. The wrapper approach identifies the best feature from the 10 optimal features for each sensor. Table 3 presents the optimal features set for each axial sensor and classification accuracy for selected optimal set.

**Figure 9:**
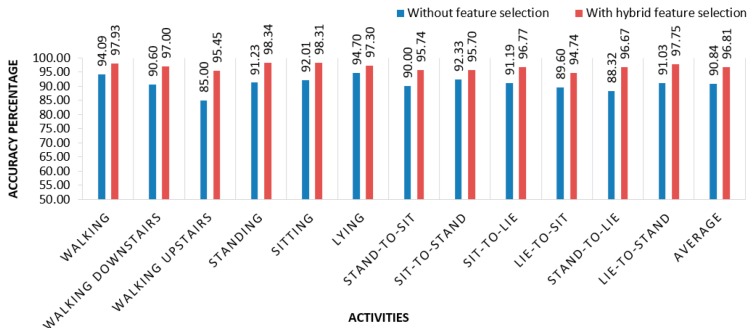
Individual activity accuracies with and without feature selection.

**Figure 10:**
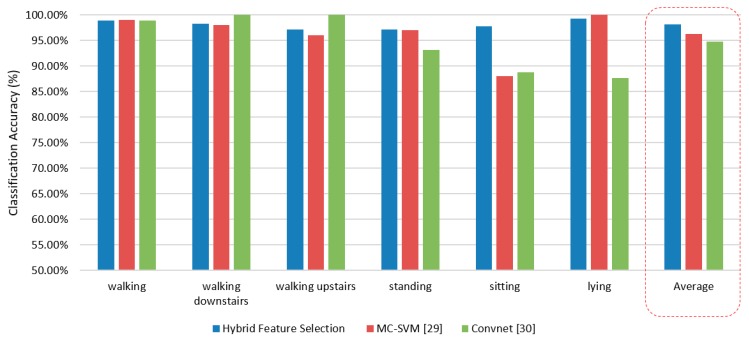
Comparison of accuracy of different classification model.

In the validation process, these best features are used to validate the overall process to check robustness our proposed model. Our proposed system works efficiently with limited computational resource and provides satisfactory activity identification. It is found that the combination of these features {A_x_ f_7_, A_x_ f_15_, A_y_ f_13_, A_z_ f_1_, G_x_ f_14_, G_z_ f_13_} produces the best result with an average accuracy of 96.81%, whereas accuracy without feature selection is 90.84%. Figure 9 depicts the accuracy of individual activity with feature selectiona and without feature selectio. In [31], the researchers used neural networks and random forests to detect human activities. In previous research, accuracy rate was below than 95% for different activities on average. Table 4 shows the confusion matrix of classification using optimal feature set. Figure 10 present the classification performance of activities using proposed feature selection model.

## 5. Additional Experimental

This research also has conducted another activity dataset by the same group of researchers of the first dataset [32]. This dataset was published with six activities rather than 12. The collection procedure and setup was same as previous research. A group of 30 people with an age range of 19–48 years were selected for data generation in this experiment. The recorded activities are walking, walking downstairs, walking upstairs, standing, sitting, and laying. Volunteers in the experiment were given a mountable a Samsung Galaxy S II smartphone on their waist. This smartphone has multiple sensors including an accelerometer and gyroscope, which were used to generate a time series of data for the experiment. Both sensors generate three-axial data series depending on the orientation of the object. Each subject did each activity two times and there was separation of 5 s between every task. The proposed hybrid optimal feature selection and multiclass support vector-based activity recognition model is implemented on this dataset in a similar fashion. The proposed model shows 98.13% accuracy. Table 5 shows the confusion matrix of proposed model on the second dataset.

Anguita et al. proposed an activity classification model using multiclass SVM and showed the result in a confusion matrix [32]. They extracted basic features including energy of different frequency bands, frequency Skewness, and angle between vectors, which in turn formulated a total of 561 features in high-dimensional features vector. The dataset was arbitrarily divided into two sets: 70% used for training and the rest was kept for testing. Multiclass support vector machine was used for classification through a One-Vs-All (OVA) approach where 10-fold cross validation procedure was applied by using the Gaussian kernel. The authors achieved 96% classification accuracy. However, this research used a high-dimensional feature vector, which demands computational overhead to model during classification process.

Ronao et al. worked on this dataset and used deep convolution neural network (convnet) for activity recognition [33]. The authors adopted a convolution process to effectively exploit the temporally local dependency of time-series signals where the pooling operation dismissed the outcome of small translations in the input. This multilayer convnet allow automatic extraction of basic features in the lower layers and complex feature in the higher layers from raw time-series sensor data. The neural network consisted of four layers and the number of feature maps from 10 to 200 in intervals of 10. They applied a greedy tuning hyperparameters for layers, feature maps, filter-size, and pooling size in the best configuration possible. Thus, they incorporated max pooling, a learning rate of 0.01, padding for ‘full’ convolution, a Gaussian initialization of U (0.05, 0.05), weight decay value of 0.00005, and momentum of 0.99. They observed the best outcome was around 95.75%. This proposed model presents a good way to use convent for activity recognition. However, the proposed model need a considerable resource consumption. To overcome the challenge, authors used a GPA unit such as NVIDIA Quadro and Intel Xeon E5-2630v2. In terms of computational overhead and classification performance, the proposed hybrid feature selection and SVM model outperform the state-of-the-art models. Figure 10 shows the comparison of accuracy of the proposed model with a state-of-the-art activity recognition model.

Win Win Myo et al. worked with this dataset as well [34]. They implemented a new method, named the cyclic attribution technique (CAT) feature selection method for identifying the human activities based on group theory and fundamental properties of the cyclic group with a binary operation involving some special properties. For classification purpose, they used an artificial neural network with a feed-forward propagation algorithm that had two hidden layers with a learning rate of 0.0005. All selected available data are divided into 70% for training set and 30% for testing. They claimed that their proposed method discovers the most important features and removes 498 features from 561 with better accuracy of 96.7% in HAR. Table 6 compares classification accuracies of different feature selection algorithms between the proposed model and other models.

## 6. Conclusions

The research presents hybrid feature selection model-based smartwatch sensor data, which robustly identify different human activities. The data are gathered from inertial sensors (accelerometer and gyroscope) mounted on the waist of the objects i.e., humans. Different activities of human were recorded by the researchers in a research lab. A total of 23 base features were applied on the dataset. A total 138 heterogeneous features are extracted from the sensors. However, as a rule of thumb, not every feature contributes in the same way for activity recognition; rather, extraneous features degrade the performance of the classifier. Thus, the proposed hybrid feature selection method containing the filter and wrapper approaches has played an important role for selecting optimal features. Finally, the selected features are used for validation test using the SVM to identify the human activities. The proposed system shows 96.81% average classification performance using optimal features, which is around 6% higher improved performance with no feature selection. The proposed model outperforms other state-of-the-art models.

This proposed system has the ability to classify given data, however integration with Internet of Things (IoT) will enable this system to use actuators and other equipment to further analyze and implement a real-world workable system. For instance, a fall of a subject would be classified as a sudden and unexpected change from their current state and through IoT, we can alert the proper authority, such as a caregiver in an old-home facility. Moreover, time-critical applications can benefit from the temporal composition of activities by predicting the activity of our subjects i.e., human beings that are vulnerable to normal environmental challenges. When we have a significant amount of data, we can predict the behavior and take actions where applicable. We believe, in many cases, the subjects in a given environment, such as elderly care or the hospital, will have a limited range of activities; based on accurately classified data, we can successfully extract enough activities and model the predicted behavior in a given context, thus helping them if there is any discrepancy.

## Figures and Tables

**Table 1:** Statistical features from sensor data.

Features	Equation	Features	Equation
Mean	$µ=\frac{1}{N}$ $\overset{N}{\underset{i=1}{\sum }}x_{i}$	Root Mean Square	$x_{\mathrm{rms}}=\sqrt{\frac{1}{N}\left(x_{1}^{2}+x_{2}^{2}\ldots +x_{n}^{2}\right)}$
Standard Deviation	$s=\sqrt{\frac{1}{N-1}\overset{N}{\underset{i=1}{\sum }}{\left(x_{i}-\bar{x}\right)}^{2}}$	Energy	$E=\overset{N}{\underset{i=1}{\sum }}{\left|x_{i}\right|}^{2}$
Median	$M=\left(\frac{\frac{n}{2}-cf}{f}\;\right)\left(w\right)+L_{m}$	SRA	$SF=µ\left(\sqrt[{2}]{abs_{X}}\right)$
Maximum	MAX(M)	Peak to Peak	PPV=MAX(M)−MIN(M)
Minimum	MIN(M)	Crest Factor	$c=\frac{\left|x_{peak}\right|}{x_{rms}}$ $=\frac{\left|\left|x\right|\right|\infty }{\left|\right|x|{|}_{2}}$
Interquartile Range	$IRQ=\frac{3}{4\left(n+1\right)}th\;term\;$ − $\frac{1}{4\left(n+1\right)}th\;term$	Impulse Factor	$i=\frac{x_{peak}}{x_{mean}}$
Correlation coefficient	$r=\frac{n\left(\sum^{\text{​}}xy\right)-\left(\sum^{\text{​}}x\right)\left(\sum^{\text{​}}y\right)}{\left[n\sum^{\text{​}}x^{2}-{\left(\sum^{\text{​}}x\right)}^{2}\right]\left[n\sum^{\text{​}}y^{2}-{\left(\sum^{\text{​}}y\right)}^{2}\right]}$	Margin Factor	$MF=\;x_{peak}/x_{sra}$
Skewness	$SV=\frac{1}{N}\overset{N}{\underset{I-1}{\sum }}{\left(\frac{x_{i-}x}{\sigma }\right)}^{3}$	Shape Factor	$SF=X_{rms}/\;µ\left(abs_{X}\right)$
Kurtosis	$\mathrm{KV}=\frac{1}{N}\overset{N}{\underset{i=1}{\sum }}{\left(\frac{x_{i}-\bar{x}}{\sigma }\right)}^{4}$	Frequency Center	$FC=\sqrt{f_{1}f_{2}}$
Cross Correlation	$\rho_{i,j}=\frac{X_{i,j}}{\sqrt{\sigma_{i}^{2}\sigma_{j}^{2}}}$	RMS Frequency	$RMS_{fr}=µ\left(\sqrt{\left({\mathrm{fr}}_{x}{}_{1}^{2}+{\mathrm{fr}}_{x}{}_{2}^{2}\ldots +{\mathrm{fr}}_{x}{}_{n}^{2}\right)}\right)$
Absolute Mean value	$A_{M}=\;µ\left(abs_{x}\right)$	Root Variant Frequency	$\sigma_{y}^{2}\left(M,T,\tau \right)=\frac{1}{M-1}\left\{\overset{M-1}{\underset{i-0}{\sum }}y_{i}^{-2}-\frac{1}{M}{\left[\overset{M-1}{\underset{I-0}{\sum }}y_{i}\right]}^{2}\right\}$
Variance	$\sigma^{2}=\left[\sum^{\text{​}}{\left(x-\;\mu \right)}^{2}\right]/N$		

**Table 2:** Original feature vector.

		Acc-x	Acc-y	Acc-z	Gyro-x	Gyro-y	Gyro-z
1	Mean	A_x_ f_1_	A_y_ f_1_	Az f_1_	G_x_ f_1_	G_y_ f_1_	G_z_ f_1_
2	Standard Deviation	A_x_ f_2_	A_y_ f_2_	Az f_2_	G_x_ f_2_	G_y_ f_2_	G_z_ f_2_
3	Median	A_x_ f_3_	A_y_ f_3_	A_z_ f_3_	G_x_ f_3_	G_y_ f_3_	G_z_ f_3_
4	Maximum	A_x_ f_4_	A_y_ f_4_	A_z_ f_4_	G_x_ f_4_	G_y_ f_4_	G_z_ f_4_
5	Minimum	A_x_ f_5_	A_y_ f_5_	A_z_ f_5_	G_x_ f_5_	G_y_ f_5_	G_z_ f_5_
6	Interquartile Range	A_x_ f_6_	A_y_ f_6_	A_z_ f_6_	G_x_ f_6_	G_y_ f_6_	G_z_ f_6_
7	Correlation coefficient	A_x_ f_7_	A_y_ f_7_	A_z_ f_7_	G_x_ f_7_	G_y_ f_7_	Gz f_7_
8	Skewness	A_x_ f_8_	A_y_ f_8_	A_z_ f_8_	G_x_ f_8_	G_y_ f_8_	G_z_ f_8_
9	Kurtosis	A_x_ f_9_	A_y_ f_9_	A_z_ f_9_	G_x_ f_9_	G_y_ f_9_	G_z_ f_9_
10	Cross Correlation	A_x_ f_10_	A_y_ f_10_	A_z_ f_10_	G_x_ f_10_	G_y_ f_10_	G_z_ f_10_
11	Mean Absolute Value	A_x_ f_11_	A_y_ f_11_	A_z_ f_11_	G_x_ f_11_	G_y_ f_11_	G_z_ f_11_
12	Variance	A_x_ f_12_	A_y_ f_12_	A_z_ f_12_	G_x_ f_12_	G_y_ f_12_	G_z_ f_12_
13	Root Mean Square	A_x_ f_13_	A_y_ f_13_	A_z_ f_13_	G_x_ f_13_	G_y_ f_13_	G_z_ f_13_
14	Energy	A_x_ f_14_	A_y_ f_14_	A_z_ f_14_	G_x_ f_14_	G_y_ f_14_	G_z_ f_14_
15	SRA	A_x_ f_15_	A_y_ f_15_	A_z_ f_15_	G_x_ f_15_	G_y_ f_15_	G_z_ f_15_
16	Peak to Peak	A_x_ f_16_	A_y_ f_16_	A_z_ f_16_	G_x_ f_16_	G_y_ f_16_	G_z_ f_16_
17	Crest Factor	A_x_ f_17_	A_y_ f_17_	A_z_ f_17_	G_x_ f_17_	G_y_ f_17_	G_z_ f_17_
18	Impulse Factor	A_x_ f_18_	A_y_ f_18_	A_z_ f_18_	G_x_ f_18_	G_y_ f_18_	G_z_ f_18_
19	Margin Factor	A_x_ f_19_	A_y_ f_19_	A_z_ f_19_	G_x_ f_19_	G_y_ f_19_	G_z_ f_19_
20	Shape Factor	A_x_ f_20_	A_y_ f_20_	A_z_ f_20_	G_x_ f_20_	G_y_ f_20_	G_z_ f_20_
21	Frequency Center	A_x_ f_21_	A_y_ f_21_	A_z_ f_21_	G_x_ f_21_	G_y_ f_21_	G_z_ f_21_
22	RMS Frequency	A_x_ f_22_	A_y_ f_22_	A_z_ f_22_	G_x_ f_22_	G_y_ f_22_	G_z_ f_22_
23	Root Variant Frequency	A_x_ f_23_	A_y_ f_23_	A_z_ f_23_	G_x_ f_23_	G_y_ f_23_	G_z_ f_23_

**Table 3:** Overall classification accuracy of final optimal features in wrapper approach in hybrid feature selection.

	Accelerometer Axial			Gyroscope Axial		
	_x_	_y_	_z_	_x_	_y_	_z_
Best Optimal feature	*f_a_x_2_* = {A_x_ f_2,_ A_x_ f_7,_ A_x_ f_9,_ A_x_ f_13,_ A_x_ f_15,_ A_x_ f_21_}	*f_a_y_9_* = {A_y_ f_2,_ A_y_ f_8,_ A_y_ f_9,_ A_y_ f_13,_ A_y_ f_15,_ A_y_ f_22_}	*f_a_z_5_* = {A_z_ f_1,_ A_z_ f_2,_ A_z_ f_7,_ A_z_ f_8,_ A_z_ f_14,_ A_z_ f_15,_ A_z_ f_21_}	*f_g_x_4_ =* {G_x_ f_2,_ G_x_ f_3,_ G_x_ f_7,_ G_x_ f_8,_ G_x_ f_13,_ G_x_ f_14,_ G_x_ f_21,_ G_x_ f_22_}	*f_g_y_7_* = {G_y_ f_2,_ G_y_ f_8,_ G_y_ f_9,_ G_y_ f_14,_ G_y_ f_15,_ G_y_ f_17,_ G_y_ f_22_}	*f_g_z_1_* = {G_z_ f_2,_ G_z_ f_7,_ G_z_ f_9,_ G_z_ f_13,_ G_z_ f_14,_ G_z_ f_15,_ G_z_ f_21_}
Overall accuracy	94.15%	92.8%	92.25%	93.15%	90.1%	92.15%

**Table 4:** Confusion matrix of classification using optimal feature set.

		Predicted Class												
		Walking	Walking Downstairs	Walking Upstairs	Standing	Sitting	Lying	Stand-to-Sit	Sit-to-Stand	Sit-to-Lie	Lie-to-Sit	Stand-to-Lie	Lie-to-Stand	Recall
**Actual class**	**walking**	189	2	2		0	0	0	0	0	0	0	0	97.93%
	**walking downstairs**	2	259	0	0	2	0	0	3	0	0	1	0	97.00%
	**walking upstairs**	5	5	252	0	0	0	0	2	0	0	0	0	95.45%
	**standing**	0	0	0	178	1	0	2	0	0	0	0	0	98.34%
	**sitting**	0	0	0	3	175	0	0	0	0	0	0	0	98.31%
	**lying**	0	0	0	3	0	180	0	0	0	0	2	0	97.30%
	**stand-to-sit**	1	0	1	0	0	0	90	0	0	0	2	0	95.74%
	**sit-to-stand**	0	0	0	0	1	0	0	89	3	0	0	0	95.70%
	**sit-to-lie**	0	0	0	0	0	1	0	0	90	0	2	0	96.77%
	**lie-to-sit**	0	0	0	0	0	0	1	1	0	90	0	3	94.74%
	**stand-to-lie**	0	0	0	0	0	1	1	0	1	0	87	0	96.67%
	**lie-to-stand**	0	0	0	0	0	0	0	0	0	2	0	87	97.75%
	**Precision**	95.94%	97.37%	98.82%	96.74%	97.77%	98.90%	95.74%	93.68%	95.74%	97.83%	92.55%	96.67%	96.81%

**Table 5:** Confusion matrix of classification for six basic activities.

		Predicted Class						
		Walking	Walking Downstairs	Walking Upstairs	Standing	Sitting	Lying	Recall
**Actual class**	**walking**	491	2	3	0	0	0	98.99%
	**walking downstairs**	4	413	3	0	0	0	98.33%
	**walking upstairs**	11	1	458	0	0	1	97.24%
	**standing**	0	0	1	517	14	0	97.18%
	**sitting**	0	0	0	11	480	0	97.76%
	**lying**	0	0	0	4	0	533	99.26%
	**Precision**	97.04%	99.28%	98.49%	97.18%	97.17%	99.81%	98.13%

**Table 6:** Classification accuracies of different feature selection algorithms.

	Hybrid Feature Selection	MC-SVM [29]	Convnet [30]	CAT Feature Selection [33]
walking	**98.99%**	99%	98.99%	89.24%
walking downstairs	**98.33%**	98%	100.00%	100.00%
walking upstairs	**97.24%**	96%	100.00%	94.52%
standing	**97.18%**	97%	93.23%	99.19%
sitting	**97.76%**	88%	88.80%	99.08%
lying	**99.26%**	100%	87.71%	99.12%
**Average**	**98.13%**	**96%**	**94.79%**	**96.86%**
